# PDCD4 Knockdown Induces Senescence in Hepatoma Cells by Up-Regulating the p21 Expression

**DOI:** 10.3389/fonc.2018.00661

**Published:** 2019-01-09

**Authors:** Jing Guo, Iwata Ozaki, Jinghe Xia, Takuya Kuwashiro, Motoyasu Kojima, Hirokazu Takahashi, Kenji Ashida, Keizo Anzai, Sachiko Matsuhashi

**Affiliations:** ^1^Division of Hepatology, Diabetology and Endocrinology, Department of Internal Medicine, Saga Medical School, Saga University, Saga, Japan; ^2^Health Administration Centre, Saga Medical School, Saga University, Saga, Japan

**Keywords:** PDCD4, p21, senescence, apoptosis, hepatoma cells

## Abstract

While the over-expression of tumor suppressor programmed cell death 4 (PDCD4) induces apoptosis, it was recently shown that PDCD4 knockdown also induced apoptosis. In this study, we examined the cell cycle regulators whose activation is affected by PDCD4 knockdown to investigate the contribution of PDCD4 to cell cycle regulation in three types of hepatoma cells: HepG2, Huh7 (mutant p53 and p16-deficient), and Hep3B (p53- and Rb-deficient). PDCD4 knockdown suppressed cell growth in all three cell lines by inhibiting Rb phosphorylation via down-regulating the expression of Rb itself and CDKs, which phosphorylate Rb, and up-regulating the expression of the CDK inhibitor p21 through a p53-independent pathway. We also found that apoptosis was induced in a p53-dependent manner in PDCD4 knockdown HepG2 cells (p53+), although the mechanism of cell death in PDCD4 knockdown Hep3B cells (p53-) was different. Furthermore, PDCD4 knockdown induced cellular senescence characterized by β-galactosidase staining, and p21 knockdown rescued the senescence and cell death as well as the inhibition of Rb phosphorylation induced by PDCD4 knockdown. Thus, PDCD4 is an important cell cycle regulator of hepatoma cells and may be a promising therapeutic target for the treatment of hepatocellular carcinoma.

## Introduction

Genetic alterations, including the activation of oncogenes and inactivation of tumor suppressor genes, are critical events in tumorigenesis (1, 2). Programmed cell death 4 (PDCD4) is considered a tumor suppressor gene (3) and is frequently down-regulated in several types of cancers, such as lung cancers (4), pancreatic cancers (5), hepatocellular carcinomas (6), colon cancers (7), skin carcinomas (8), invasive ductal breast carcinomas (9), and gliomas (10). The PDCD4 expression is decreased in the nuclei and increased in the cytoplasm in carcinoma cells compared with normal cells in stomach cancer tissues (11). Indeed, the loss of PDCD4 expression has been strongly implicated in the development and progression of several types of human cancers (4–10).

PDCD4 was first isolated from a human glioma complementary DNA (cDNA) library and localized to chromosome 10q24 (12, 13). It contains two MA-3 domains that are homologous to the M1 domain in the eukaryotic translation initiation factor 4G (eIF4G), which interacts with eukaryotic translation initiation factor 4A (eIF4A) to form the cap-dependent translation initiation factor complex eukaryotic translation initiation factor 4 F (eIF4F) and is thought to be involved in protein-protein interactions (14, 15). PDCD4 interacts with eIF4A via its MA-3 domains to control protein synthesis. Previous studies have shown that PDCD4 inhibits the interaction between eIF4A and eIF4G as well as eIF4A's RNA-helicase activity (16). Furthermore, PDCD4 also inhibits the transactivation of activator protein 1 (AP-1) by inhibiting the expression of the up-stream kinase of c-Jun mitogen-activated protein kinase kinase kinase kinase 1 (MAP4K1) (17). It has been shown that PDCD4 can inhibit tumor cell invasion and metastasis by suppressing AP-1-dependent transcription (17, 18). In addition, PDCD4 can also inhibit β-catenin activation by stabilizing E-cadherin and supress cell invasion (19). It has been reported that PDCD4 expression is increased during apoptosis (20, 21) and decreased by treatment with interleukin-2 or interleukin-15 (22) as well as mitogens, such as a serum (23), epidermal growth factor (EGF) and 12-O-tetradecanoylphorbor-13-acetate (TPA) (24).

Previous experiments have reported that PDCD4 over-expression induced by transforming growth factor-β1 (TGF-β1) led to Huh7 hepatoma cell apoptosis by activating Bax, releasing cytochrome c from the mitochondria and activating caspase 9, caspase 8 and caspase 3; this also occurred in cells transfected with PDCD4-plasmids (6). PDCD4 was shown to repress the transcription of the mitosis-promoting factor cyclin-dependent kinase CDK1/cdc2 via the up-regulation of p21 (Waf1/Cip1), which resulted in the suppression of retinoblastoma protein (Rb) activation and cell growth in Bon-1 carcinoid cells (25).

The siRNA-mediated knockdown of PDCD4 expression was shown to up-regulate the expression of p21 (Waf1/Cip1) and several other p53-dependent genes in HeLa cells (26). It was recently reported that apoptosis inducers, such as staurosporine (STS), antagonizing antibody to Fas (CH11), etoposide (VP16) and cycloheximide (CHX), down-regulate the PDCD4 expression; the loss of PDCD4 may also induce apoptosis by promoting pro-caspase 3 translation and up-regulating caspase 3 activity (27). In contrast, PDCD4 knockdown promotes colon HT29 cell proliferation and up-regulates cyclin D1 expression by activating Akt-NF-kB signaling (28). PDCD4 knockout mice grew to adulthood, although they had shorter lifespans than wild-type mice due mostly to the development of B cell lymphoma (29). These results indicate that PDCD4 is not essential for cell proliferation and development, although the roles and functions of PDCD4 in cell proliferation and apoptosis appear to be complicated.

To elucidate the effect of PDCD4 knockdown on the cell cycle in hepatoma cells and gain further insights into PDCD4 function, we treated cells with PDCD4-specific siRNAs and investigated the expression of factors involved in cell cycle control in hepatoma cells.

## Materials and Methods

### Cell Lines and Cell Cultures

The human hepatocellular carcinoma-derived cell lines HepG2 and Huh7 were obtained from the Japanese Cancer Research Resources Bank (Osaka Japan), and Hep3B was obtained from the American Type Culture Collection (ATCC, Manassas, VA, USA). The cells were cultured and maintained in Dulbecco's modified Eagle's medium (DMEM; Sigma-Aldrich, St. Louis, MO, USA) containing 10% fetal bovine serum (FBS) and 100 μg/ml penicillin and streptomycin in 5% CO2 at 37°C. HeLa cells were laboratory stock and cultured in DMEM with 5% FBS.

### Antibodies

An anti-PDCD4 antibody was prepared by immunizing rabbits with a synthetic peptide corresponding to the N-terminal amino acid sequence (6). The antibody against CDK1 (cdc2) was obtained from Santa Cruz Biotechnology (Dallas, TX, USA). The antibody against p16 was obtained from Abcam (Cambridge, UK). Antibodies against β-actin, Rb, phospho-Rb(807/811), phospho-Rb(780), p53, p27, p21, p18, CDK2, CDK4, CDK6, cyclin D1, caspase 3, and caspase 9 were purchased from Cell Signaling Technology (Beverly, MA, USA). The antibodies were used according to the protocols provided by companies.

### siRNA-Mediated Knockdown of PDCD4, p53, p21, and p27

The cells were used for transfection when they were 90% confluent in 100-mm dishes. siRNA transfection was performed using Lipofectamine RNAiMAX (Life Technologies, Rockville, MD, USA) and a reverse transfection method for HepG2, Hep3B, and HeLa cells and a forward transfection method for Huh7 cells, according to the manufacturer's protocols. The sequences for the PDCD4-specific siRNA p1 and p2 were 5′-GCGGAAAUGUUAAGAGAUU-3′ and 5′-GCACAACUGAUGUGGAAAA-3′, respectively (KOKEN CO., LTD, Tokyo, Japan). The sequence for the PDCD4-specific siRNA k603 was 5′-GUGUUGGCAGUAUCCUUAG-3′ (26), which was prepared by Hokkaido System Science Co., LTD (Hokkaido, Japan). The p53-specific siRNA (SI00011655), p21-specific siRNA (SI00604898), p27-specific siRNA (SI02621990) and Allstar negative control siRNA (1027281) were obtained from Qiagen (Heiden, Germany). The cells were collected for a Western blot analysis at 24, 48, 72, 96, and 120 h after transfection.

### Cell Growth Assays

siRNA transfection was performed using the forward method for Huh7 cells and the reverse method for HepG2, Hep3B, and HeLa cells, as described above. Huh7 cells were seeded at 1 × 10^4^ cells/well in 24-well plates. After culturing for 2 days, the cells were transfected with PDCD4-specific siRNAs or negative control siRNA. Approximately 2 × 10^4^ HepG2 and Hep3B cells and ~1 × 10^4^ HeLa cells were seeded in 24-well plates for continued culturing after treatment with siRNA via the reverse method. At the culture times indicated in the figures, the cells were trypsinised and counted under a microscope.

### Western Blot Analyses

The collected cells were extracted by sonication in lysis buffer containing 50 mM Tris (pH 6.8), 2.3% sodium dodecyl sulfate (SDS) and 1 mM phenylmethylsulfonyl fluoride (PMSF). The cell debris was eliminated by centrifugation at 12,000 *g* for 10 min, and the supernatant was collected. Protein amounts were determined with a *DCTM* protein assay kit (Bio-Rad, Hercules, CA, USA) using bovine serum albumin as the standard by the Lowry method. Protein (15–30 μg) from each sample was mixed with SDS loading buffer, separated by SDS polyacrylamide gel electrophoresis, and transferred to a polyvinylidene difluoride (PVDF) membrane (Bio-Rad). The membrane was blocked via incubation overnight at 4 °C in phosphate-buffered saline (PBS) containing 0.1% Tween 20 and 7% skim milk and then incubated with the primary antibody with shaking for 1 h at room temperature or overnight at 4°C. After washing 5 times with PBS containing 0.1% Tween 20, the specific bands were visualized by further incubation with horseradish peroxidase (HRP)-conjugated second antibody followed by enhanced chemiluminescence detection using the ECL system (Amersham, Buckinghamshire, UK) according to the manufacturer's instructions. For the detection of phospho-Rb (807/811) and phospho-Rb (780), Tris-buffered saline (TBS) was used instead of PBS. The rabbit polyclonal anti-β-actin antibody was used as a control. The stained membrane was exposed to Fuji Medical X-ray film (Tokyo, Japan) and the specific protein bands were determined with the Image J software program (https://imagej.nih.gov/ij/) and normalized to β-actin.

### Quantitative Real-Time Reverse Transcription Polymerase Chain Reaction (RT-PCR)

Total RNA was isolated from cells using RNAiso Plus (Takara, Kusatsu, Japan) and reverse transcribed to cDNA using a High Capacity cDNA Reverse Transcription Kit (Thermo Fisher, Waltham, MA, USA) according to the manufacturer's instructions. Real-time PCR using SYBR Select Master Mix (Thermo Fisher) was performed on a StepOnePlus system (Applied Biosystems) according to the manufacturer's instructions. The primers of GAPDH, PDCD4, p21, p27, and p18 were synthesized by Hokkaido System Science Co., LTD. (Hokkaido, Japan). The sequences of primers were as follows: GAPDH forward (F) 5′-GTCTCCTCTGACTTCAACAGCG-3′ and reverse (R) 5′-ACCACCCTGTTGCTGTAGCCAA-3′; PDCD4, F 5′-ATGAGCAGATACTGAATGTAAAC-3′and R 5′-CTTTACTTCCTCAGTCCCAGCAT-3′; p21, F 5′-TCTTGTACCCTTGTGCCTCG-3′ and R 5′-ATCTGTCATGCTGGTCTGCC-3′; p27, F 5′-GACTATCTGCTGCGCGGTTA-3′ and R 5′-TCGAGTTCCTGACAAGCCAC-3′; p18, F 5'-GGAGTTCCTGGTGAAGCACA-3' and R 5′-CCCATAGAGCCTGGCCAAAT-3′. Data were analyzed using the comparative Ct (ΔΔCt) method, and the expression of target genes was normalized to GAPDH. Each experiment was performed with three replicates.

### The Terminal Deoxynucleotidyl Transferase Dutp Nick End Labeling (TUNEL) Assay

The TUNEL assay was performed using a kit purchased from MBL (Nagoya, Japan) according to the manufacturer's protocol. TUNEL-positive nuclei were visualized by FITC-avidin, and the specimens were counter-stained with Hoechst 33342. The positive cells were then counted under a fluorescent microscope. The data are represented as the average number of apoptotic cells obtained from three independent experiments.

### Fluorescence-Activated Cell Sorting (FACS) Analyses

HepG2, Huh7, and Hep3B cells were transfected with PDCD4-specific siRNAs (p2 or k603) or negative control siRNA as previously described. The cells were plated at a density of 1 × 10^5^ cells/35-mm dish, cultured for 1, 2, 3, 4, and 5 days, trypsinised, and collected by centrifugation. The cell pellet was washed twice with PBS, suspended in 70% ethanol and maintained for 2 h at 4°C. After washing twice with PBS, the cells were suspended in PBS containing RNase (0.1 mg/ml) and PI solution (50 μg/ml) and incubated for 30 min at room temperature. The cells were then analyzed with a FACS cytofluorometer (BD FACS VerseTM), and the percentage of cells in the different cell cycle phases were determined.

### The β-Galactosidase Activity Assay

Cellular senescence was assessed using the β-galactosidase staining kit (Cell Signaling) following the manufacturer's protocols. Cells treated with siRNAs were cultured in 35-mm dishes for 24, 72, 96, and 120 h. After washing with PBS, the cells were fixed with 10% formalin solution for 15 min, stained for β-galactosidase activity using the staining solution overnight at 37°C, and counterstained with Kernechtrot (Sigma-Aldrich). Stained cells were viewed under a microscope and photographed. The data were expressed as the percentage of β-galactosidase-positive cells.

### Statistical Analyses

Differences were determined using Student's *t*-test, and *p* < 0.05 was considered significant. All the experiments were performed at least in triplicate. Data are shown as the mean ± standard deviation (SD).

## Results

### siRNA-Mediated Knockdown of PDCD4 Suppressed Cell Growth

**Figure 1:**
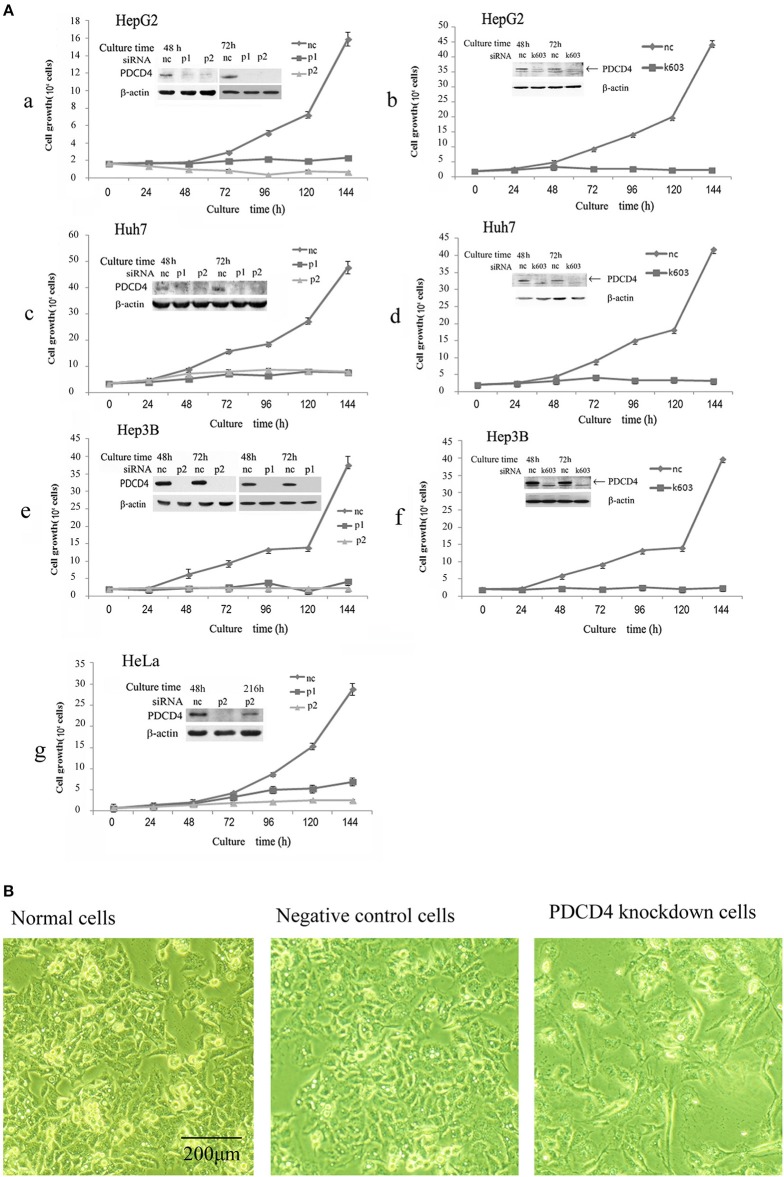
siRNA-mediated PDCD4 knockdown suppressed cell growth and induced morphological changes. **(A)** PDCD4 knockdown suppressed cell growth of HepG2 **(a,b)**, Huh7 **(c,d)**, Hep3B **(e,f)**, and HeLa **(g)** cells. Cells were transfected with three types of PDCD4-specific siRNAs: p1 or p2 **(a,c,e,g)** and k603 **(b,d,f)**, and a negative control siRNA (nc). Cell growth was analyzed by counting the cell numbers under a microscope at different time points, as indicated in the figures. **(B)** Phase-contrast images of the normal (left), negative control siRNA-treated (middle) and p2 siRNA-mediated PDCD4 knockdown HepG2 cells (right) at 48 h after siRNA treatment.

It was previously reported that the loss of PDCD4 induced apoptosis in HeLa cells and C2C12 myoblasts (27) but promoted cell proliferation in HT29 colon tumor cells (28). To assess whether the loss of PDCD4 promotes or suppresses hepatoma cell growth, HepG2 (wild-type p53), Huh7 (mutant p53 and p16-deficient) and Hep3B (p53- and Rb-deficient) hepatoma cells were treated with three types of PDCD4-specific siRNAs (p1, p2, and k603). HeLa cells were also treated similarly to the hepatoma cells as a control. As shown in Figure 1A, there were significantly fewer PDCD4 knockdown HepG2 cells than negative control cells (Figures 1Aa,1Ab). Similar results were also observed in the Huh7 (Figures 1Ac,1Ad) and Hep3B (Figures 1Ae,1Af) as well as HeLa (Figure 1Ag) cells. The results indicated that PDCD4 knockdown suppressed hepatoma cell growth. We also observed morphological changes in the PDCD4 knockdown cells that showed an enlarged and flat shape (Figure 1B, right panel) compared to the non-treated cells and negative control siRNA-treated cells (Figure 1B, left and middle panels, respectively).

In this study, we tested three kinds of PDCD4-specific siRNAs: p1, p2, and k603. p2 and k603 efficiently suppressed the PDCD4 expression and cell growth, while the knockdown efficiency of p1 was not constant and often significantly reduced compared with the other siRNAs. Therefore, p2 and k603 were used for the subsequent experiments.

### PDCD4 Knockdown Suppressed Rb and Rb Phosphorylation and Modulated the Expression of CDKs

**Figure 2:**
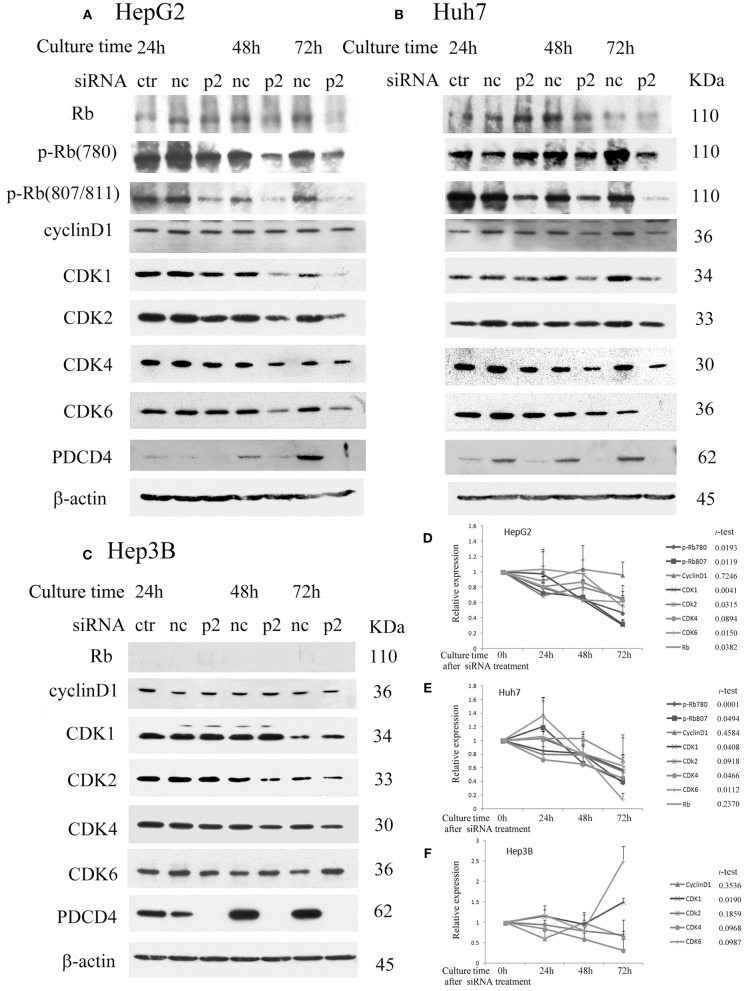
PDCD4 knockdown suppressed Rb and p-Rb and modulated the expression of CDKs. PDCD4 knockdown suppressed the expression of Rb, CDK1, CDK2, CDK4, CDK6, and Rb phosphorylation in **(A)** HepG2 (wild-type p53) and **(B)** Huh7 (mutant p53 and p16-deficient). **(C)** The expression of CDK1 significantly up-regulated while the modulations of CDK2, CDK4, and CDK6 were not significant in Hep3B (p53- and Rb-deficient) cells. Protein extracts from untreated control cells (ctr), negative control siRNA-treated cells (nc) and PDCD4-specific p2 siRNA-treated (p2) hepatoma cells were analyzed by Western blotting at 24, 48, and 72 h of culture after siRNA treatment using specific antibodies against the components indicated in the figures. These experiments were independently repeated three times, and similar results were obtained. Each band was determined and normalized to β-actin. The data in **(D)** HepG2, **(E)** Huh7, and **(F)** Hep3B represent the mean ± SD obtained from the respective experiments. The *t*-test shows the *p*-value between negative control siRNA and PDCD4-specific siRNA treated cells at 72 h of culture.

To determine the effects of PDCD4 knockdown on cell cycle regulators in hepatoma cells, we investigated the expression of retinoblastoma protein (Rb), phospho-Rb (p-Rb) and its controller cyclin-dependent kinases (CDKs) by Western blotting of the p2 and k603 PDCD4-specific siRNAs-treated cells. As shown in Figures 2A,D and Figure S1A, Rb phosphorylation was drastically down-regulated at both Ser780 and Ser807/811 in the PDCD4 knockdown HepG2 cells compared to the negative control cells. Similar results were also observed in Huh7 cells (Figures 2B,E and Figure S1B). The expression of Rb in both cell lines was also down-regulated after PDCD4 knockdown, but the decline was less than that of p-Rb (Figures 2A,B). The p1 PDCD4-specific siRNA was also tested. In the case of p1 siRNA, the PDCD4 knockdown was so often incomplete and the Rb-phosphorylation was not sufficiently suppressed compared with that with p2 siRNA (Figure S1D). Therefore, we have not used p1 siRNA after that. The expression of all of the CDKs was down-regulated in the PDCD4 knockdown HepG2 and Huh7 cells (Figures 2A,B,D,E, and Figures S1A,B). The cyclin D1 expression tended to be suppressed in the PDCD4 knockdown cells compared to the cells treated with the negative control siRNA, although the differences were not significant (Figure 2 and Figure S1). In contrast, the Rb band was not detected in Hep3B cells, which is consistent with previous results showing that these cells are Rb-deficient (30). The CDK expression patterns were different and less affected in the PDCD4 knockdown Hep3B cells than in the HepG2 and Huh7 cells (Figures 2C,F and Figure S1C). The CDK1 expression was up-regulated significantly with both p2 and k603 siRNA, but that of CDK2, CDK4 and CDK6 was not modulated significantly in both cases of p2 and k603 siRNA treated Hep3B cells (Figures 2C,F and Figure S1C).

The results suggest that PDCD4 knockdown might interrupt the cell cycle by suppressing Rb phosphorylation, at least partially by down-regulating the expression of CDKs.

### PDCD4 Knockdown Up-Regulated the Expression of p21

**Figure 3:**
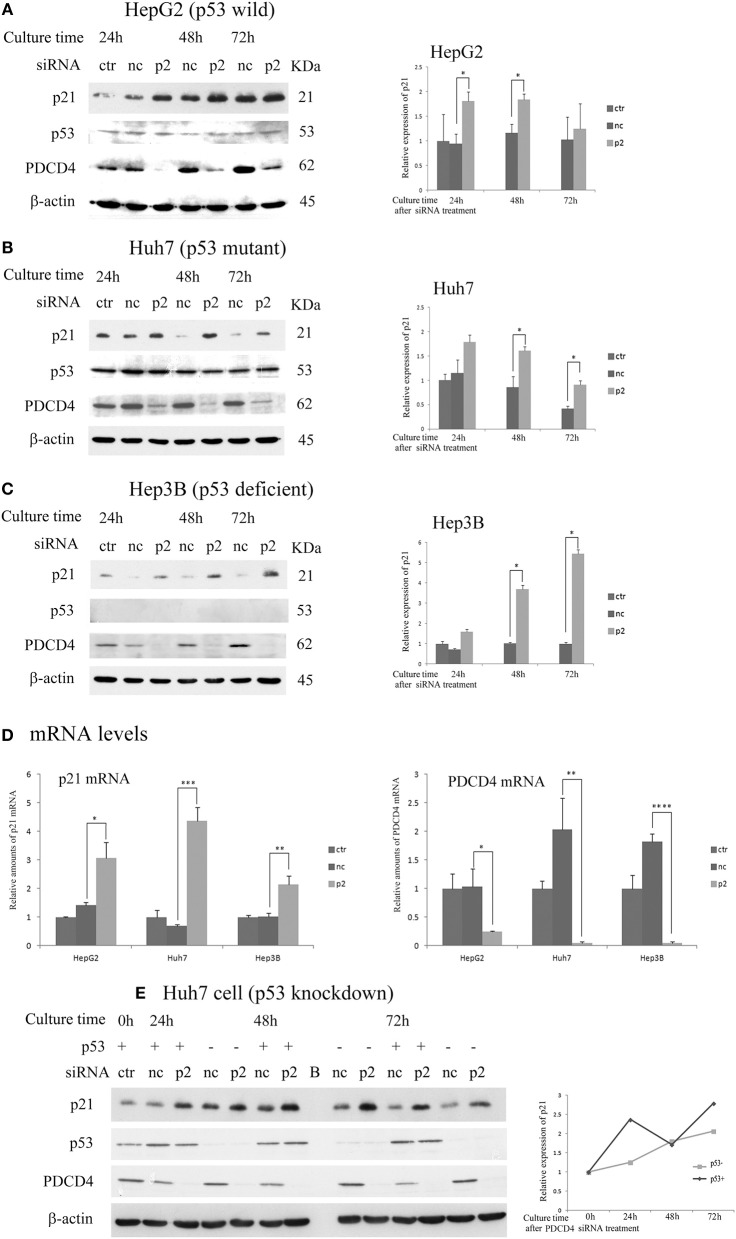
PDCD4 knockdown up-regulated the expression of p21 in HepG2, Huh7, and Hep3B cells. The experiments were performed as in Figure 2. A Western blot analysis of the untreated cells (ctr), negative control siRNA-treated cells (nc), and p2 siRNA-mediated PDCD4 knockdown cells (p2), were performed using antibodies against p21, p53, PDCD4 and β-actin. **(A–C)** Western blotting (Left) and a diagram of the relative p21 expression obtained from the Western blot (Right) of cell extracts obtained from **(A)** HepG2, **(B)** Huh7 and **(C)** Hep3B cells. The experiments were independently repeated 3 times, and the p21 expression was normalized to β-actin. The data in the diagrams represent the mean ± SD obtained from the experiments. *t*-test: * *p* < 0.05. **(D)** A real time RT-PCR analysis of p21 in PDCD4 knockdown HepG2, Huh7, and Hep3B cells. The cells were treated with negative control siRNA (nc) or PDCD4-specific p2 siRNA (p2) and cultured for 48 h. The experiments were repeated independently 3 times. Data were expressed as the mean ± SD obtained from the experiments. *t*-test: *p < 0.05; **p < 0.005;***p < 0.0005; ****p < 0.00005. **(E)** The p21 expression in p53 knockdown Huh7 cells. The cells were transfected with negative control siRNAs (p53+) or p53-specific siRNAs (p53-). After 24 h, the transfected cells were again treated with negative control siRNA (nc) or PDCD4-specific p2 siRNA (p2). (left panel) Western blotting of Huh7 cells using antibodies against PDCD4, p21, p53 and β-actin at 24, 48, and 72 h after PDCD4 knockdown. (Right) The relative p21 expression in Huh7 cells with and without p53 obtained from the left panel is shown. The amounts of p21 were normalized to β-actin, and the amounts of p21 in the PDCD4 knockdown cells (p2) were expressed as the relative amount compared to the negative control siRNA-treated cells (nc).

Next, the expression of p21 (Waf1/Cip1) and p27 (Kip), which are CDK inhibitors, was assayed after the p2 or k603 siRNA-mediated PDCD4 knockdown in these three hepatoma cell lines. The p21 protein levels were up-regulated in HepG2 and Huh7 cells soon after PDCD4 knockdown (Figures 3A,B and Figures S2A,**B**), as well as the mRNA levels (Figure 3D and Figure S2D). The level of p53, which stimulates the p21 expression, was not changed markedly in either HepG2 or Huh7 cells (Figures 3A,B and Figures S2A,B). Despite lacking the p53 gene, both the p21 protein and mRNA levels were also increased in PDCD4 knockdown Hep3B cells (Figures 3C,D and Figures S2C,D). These results suggest that a p53-independent p21 expression pathway was activated in these hepatoma cells. To confirm this, p53 knockdown Huh7 cells were treated with PDCD4-specific p2 siRNAs. As shown in Figure 3E, the p21 levels were significantly increased after PDCD4 knockdown in the p53 knockdown Huh7 cells. Similar results were obtained using HepG2 cells (Figure S2E). These results indicate that PDCD4 knockdown induced the p21 expression in a p53-independent manner.

**Figure 4:**
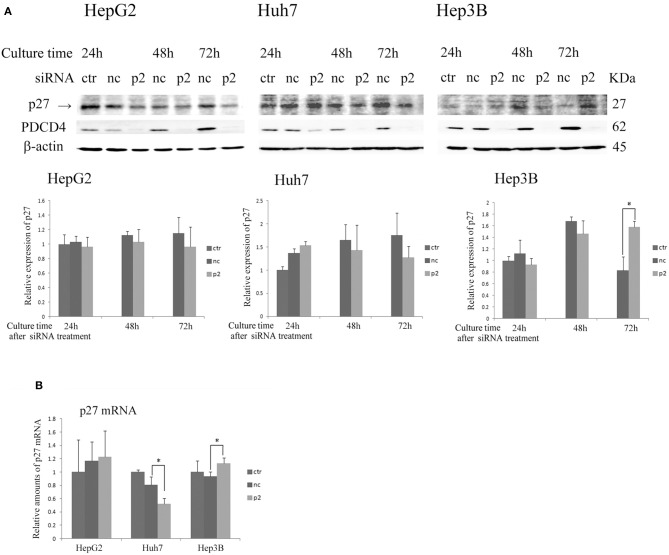
The p27 (Cip/Kip) expression was changed only slightly or down-regulated in HepG2 and Huh7 but was up-regulated in Hep3B cells by p2 siRNA-mediated PDCD4 knockdown**(A)** The hepatoma cells HepG2, Huh7, and Hep3B were treated with negative control siRNA (nc) or PDCD4-specific p2 siRNA (p2) and then subjected to a Western blot analysis using p27-, PDCD4-, and β-actin-specific antibodies at the culture time indicated in the figures. ctr, control cells without siRNA treatment. The experiments were repeated at least three times, and similar results were obtained each time. The data in the diagrams represent the mean ± SD obtained from the experiments. *t*-test: * *p* < 0.05. →, the p27 band was identified by p27 knockdown using a specific siRNA. **(B)** A real time RT-PCR analysis of p27 in PDCD4 knockdown HepG2, Huh7, and Hep3B cells. The cells were treated with negative control siRNA (nc) or PDCD4-specific p2 siRNA (p2) and cultured for 48 h. The experiments were repeated independently 3 times. Data were expressed as the mean ± SD obtained from the experiments. *t*-test: *p < 0.05.

As shown in Figure 4 and Figure S3, the expression of p27 was not changed or down-regulated by PDCD4-specific siRNA treatments at both levels of protein and mRNA in HepG2 and Huh7 cells, while the same treatment instead up-regulated the p27 protein level as well as the mRNA expression in Hep3B cells.

The results indicate that PDCD4 knockdown mediated by specific siRNAs inhibits cell cycle progression at least in part by suppressing the activities of CDKs by up-regulating the CDK inhibitor p21, which leads to Rb phosphorylation inhibition.

### Modulation of INK4 p16 and p18 Expression in PDCD4 Knockdown Cells

**Figure 5:**
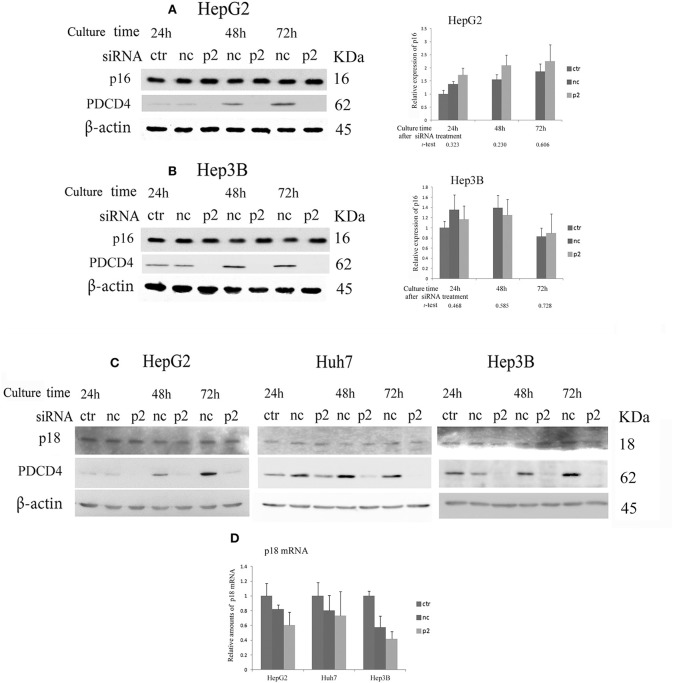
The expression of the INK4 inhibitors p16 and p18 in PDCD4 knockdown cells. Cells were treated with negative control siRNA (nc) or PDCD4-specific p2 siRNA (p2) and subjected to a Western blotting analysis with p16-, p18-, PDCD4-, and β-actin-specific antibodies after culturing for the times indicated in the figures. **(A,B)** A Western blot analysis (Left) and diagrams of the relative p16 expression (Right) obtained from the Western blot in **(A)** HepG2 and **(B)** Hep3B cells. The experiments were repeated independently three times. The data in the diagrams represent the mean ± SD obtained from the three experiments. (*t*-test: *p*-values determined by Student's *t*-test from the p16 amounts in p2 and nc). The *p*-values show that the difference in the p16 expression between PDCD4 knockdown cells and negative control cells was not significant. **(C)** The p18 levels were not markedly changed by PDCD4 knockdown in HepG2, Huh7, and Hep3B cells. These experiments were repeated at least three times, and similar results were obtained each time. ctr, control cells without treatment with siRNAs; nc, negative control siRNA-treated cells; and p2, PDCD4-specific p2 siRNA-treated cells. **(D)** A real time RT-PCR analysis of p18 in PDCD4 knockdown HepG2, Huh7, and Hep3B cells. The cells were treated with negative control siRNA (nc) or PDCD4-specific p2 siRNA (p2) and cultured for 48 h. The experiments were repeated independently 3 times.

When hepatoma cells were treated with PDCD4-specific siRNAs, the cells showed enlarged and flattened morphological changes that are usually observed in senescent cells (Figure 1B). These phenomena indicated that the hepatoma cells with PDCD4 knockdown might be undergoing differentiation or cellular senescence. Therefore, we assayed CDK4/6 inhibitors, including p16 INK4A, which is associated with replicative senescence (31), and p18 INK4C, which is associated with differentiation (32). The significant modulation of p16 expression was not observed with both p2 and k603 siRNA treatments at least until 72 h culture (Figures 5A,B and Figure S4B) compared to the negative control siRNA treatments in HepG2 and Hep3B cells. The p16 band was not detected in p16-deficient Huh7 cells (33). The p18 expression was little changed at both protein and mRNA levels in HepG2, Huh7, and Hep3B cells under PDCD4 knockdown using both p2 (Figures 5C,D) and k603 siRNAs (Figures S4C,D).

### PDCD4 Knockdown May Induce Cell Death Through Different Mechanisms in p53-Wild-Type HepG2 and p53-Deficient Hep3B Cells

**Figure 6:**
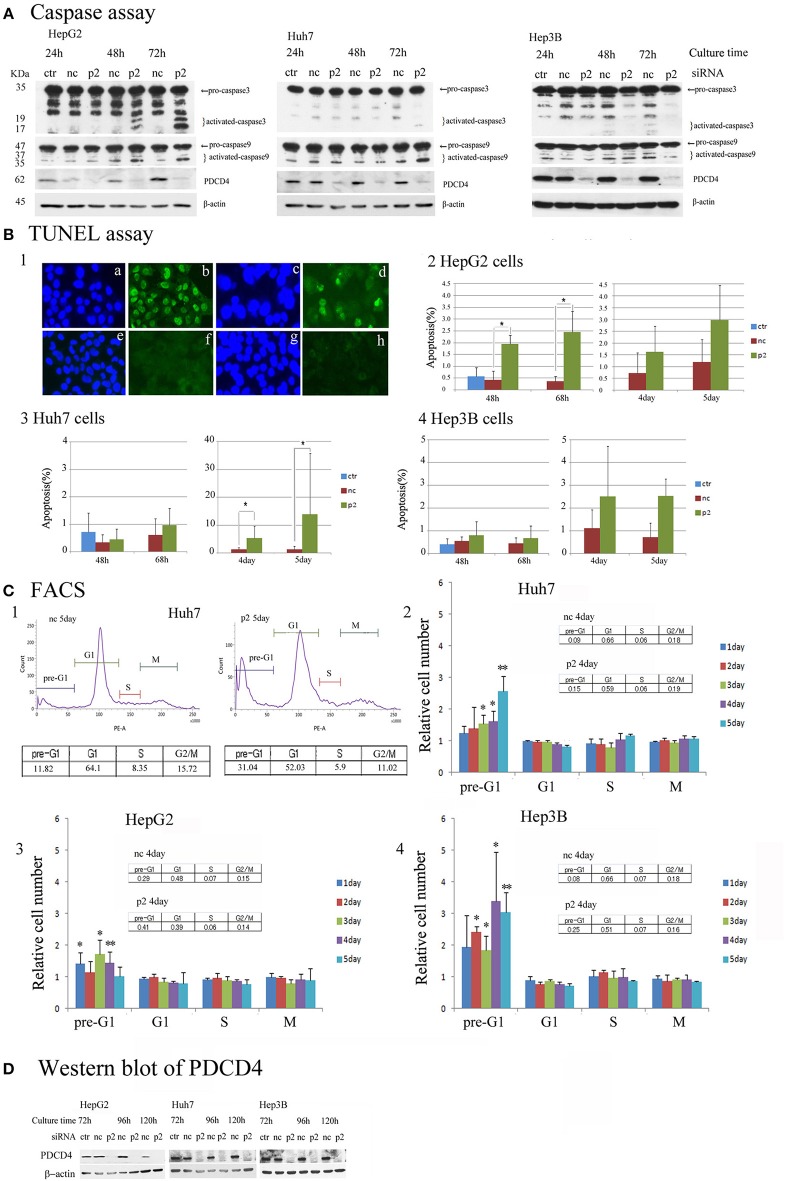
Apoptosis induced by PDCD4 knockdown in HepG2, Huh7 and Hep3B hepatoma cells. **(A)** Caspase assays. The experiments were performed as in Figure 2. An immunoblot analysis was performed with antibodies recognizing caspase 3 and caspase 9 for cell extracts of the untreated cells (ctr), negative control siRNA-treated cells (nc) and PDCD4 specific p2 siRNA-treated cells (p2) in HepG2, Huh7, and Hep3B cells. These experiments were repeated two times, and similar results were obtained in both cases. **(B)** The TUNEL assay. HepG2, Huh7, and Hep3B cells were treated with negative control siRNA (nc) and PDCD4-specific p2 siRNA (p2) and subjected to a TUNEL assay at the culture times indicated in the figures after siRNA transfection. TUNEL-positive cell nuclei were stained with FITC-avidin and the nuclei were visualized by Hoechst staining. Panel 1 shows **(a,c,e,g)** Hoechst staining and **(b,d,f,h)** FITC-images of **(a,b)** HepG2 cells that were treated with DNase as a positive control, **(c,d)** PDCD4 knockdown cells, **(e,f)** untreated control cells and **(g,h)** negative control siRNA-treated cells. Panels 2, 3 and 4 show the amount of apoptotic cells (%) in the control cells without siRNA treatment (ctr), negative control siRNA-treated cells (nc), and PDCD4-specific p2 siRNA-treated cells (p2) in the (2) HepG2, (3) Huh7, and (4) Hep3B. Positive cells were counted under a fluorescent microscope, and the data are shown as the mean ± SD obtained from three independent experiments. Thousands of cells were counted in each experiment. *t*-test: *p < 0.05. **(C)** The FACS analysis. HepG2, Huh7, and Hep3B cells were transfected with PDCD4-specific p2 siRNA (p2) and negative control siRNA (nc) as described in the methods. After 1, 2, 3, 4, and 5 days of culture, the cells were collected and stained with propidium iodide (PI). DNA content was then analyzed by FACS. For each sample about 5,000 cells were analyzed. (1) FACS of a negative control siRNA-transfected (nc) and PDCD4-specific p2 siRNA-transfected (p2) Huh7 cell sample at 5 days of culture after siRNA transfection. (2, 3, and 4) The culture time-dependent changes in the cell population at the pre-G1, G1, S and G2/M phases of the cell cycle in (2) Huh7, (3) HepG2, and (4) Hep3B cells are represented as the ratio of PDCD4 siRNA-transfected cells to negative control siRNA-transfected cells. The experiments were independently repeated at least three times, and the data represent the mean ± SD obtained from the experiments. *t*-test between negative control and PDCD4 knockdown cells: *p < 0.05; **p < 0.005. **(D)** PDCD4 levels analyzed by Western blot in PDCD4 knockdown cells.

To determine the mechanism underlying the growth suppression in hepatoma cells via PDCD4 knockdown, the pro-apoptotic markers caspase 3 and caspase 9 were assayed by a Western blotting analysis. PDCD4 knockdown activated caspase 3 and caspase 9 in HepG2 cells with wild-type p53, although the active forms of the caspases were detected slightly and not at all in the Huh7 with mutant p53 and Hep3B with null p53 cells (Figure 6A and Figure S5A). Cells cultured for 5 days after PDCD4 knockdown were also assayed, and caspase 3 activation was found to be up-regulated in HepG2 cells but not to a significant degree in Huh7 and Hep3B cells (Figure S5A4).

In the TUNEL assays, significant numbers of positive cells were observed after 2 days' culture, and positive cells were observed until at least 2 days' culture in the PDCD4 knockdown HepG2 cells containing wild-type p53 (Figures 6B1,2 and Figure S5B). In contrast to HepG2 cells, TUNEL-positive cells were only faintly detectable at Day 3 in the PDCD4 knockdown Huh7 and Hep3B cells (Figures 6B3,4 and Figure S5B). However, the number of TUNEL-positive cells among the PDCD4 knockdown Huh7 cells increased after 4 days' culture, and more than 10% of the cells were TUNEL-positive on Day 5 of culture (Figure 6B3 and Figure S5B) despite a lack of caspase 3 activation. In the p53-deficient Hep3B cells, there were fewer TUNEL-positive cells than among HepG2 or Huh7 cells (Figure 6B4 and Figure S5B), while pre-G1 cells were observed from the first day of culture after PDCD4 knockdown, as shown by a FACS analysis (Figure 6C4 and Figure S5C). Furthermore, the FACS analysis revealed that there was no accumulation of cells in any specific cell cycle phase, except for an increase in the pre-G1 cell population, among the PDCD4 knockdown hepatoma cells (Figure 6C and Figure S5C). These results indicated that PDCD4 knockdown facilitates apoptosis mostly via wild-type p53 activation and that different cell death mechanisms other than apoptosis might occur in the p53-mutant and p53-null hepatoma cells.

### Senescence Was Induced in the PDCD4 Knockdown Hepatoma Cells as Judged by the β-Galactosidase Activity

**Figure 7:**
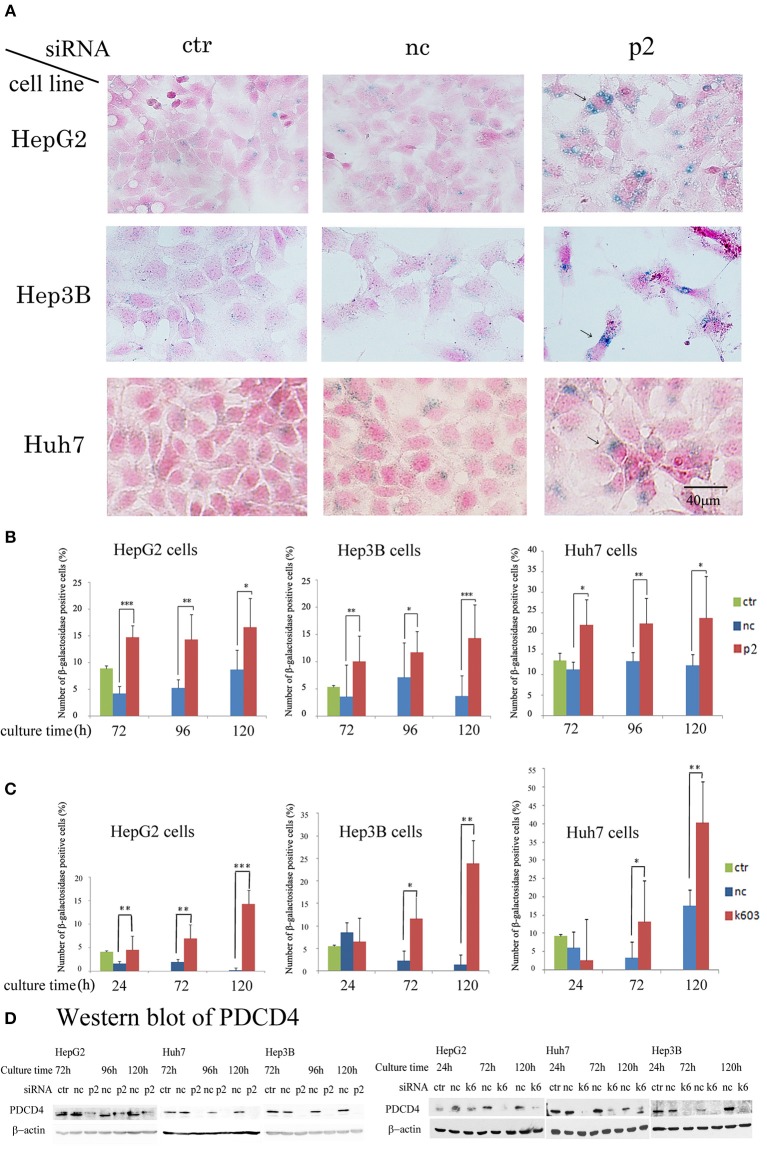
PDCD4 knockdown induced replicative senescence. **(A)** β-galactosidase staining patterns in the untreated control cells (ctr), negative control siRNA-treated cells (nc), and PDCD4-specific p2 siRNA-treated cells (p2) in HepG2, Hep3B, and Huh7 cells at 4 day of culture after the siRNA treatments. The cells with arrows are enlarged and show morphological changes and β-galactosidase positivity. **(B)** The number of β-galactosidase-positive HepG2, Hep3B, and Huh7 cells after PDCD4 knockdown with p2 siRNA (p2), treatment with negative control siRNA (nc) and no treatment (ctr). **(C)** The number of β-galactosidase-positive HepG2, Hep3B, and Huh7 cells after PDCD4 knockdown with k603 siRNA (k603), treatment with negative control siRNA (nc) and no treatment (ctr). The data represent the average cell number of three different fields, and thousands of cells were counted in each field. These experiments were repeated independently three times, and similar results were obtained each time. A representative result from the experiments is shown in this figure. *t*-test: *p < 0.05; **p < 0.005; and ***p < 0.0005. **(D)** PDCD4 levels analyzed by Western blot in PDCD4 knockdown cells.

Our data suggested that the PDCD4 knockdown cells might have become senescent. To test this, the activity of β-galactosidase, a marker of cell senescence, was assayed. As shown in Figure 7, β-galactosidase activity was induced in the PDCD4 knockdown HepG2, Huh7, and Hep3B cells.

In the Huh7 cells, which were deficient in p16 expression, the number of β-galactosidase-positive cells among the control siRNA-treated cells was much higher than that among the HepG2 and Hep3B cells (Figure 7). However, the staining intensity was higher in the PDCD4 knockdown cells than in the negative control cells (Figure 7A), and there were significantly more positive cells observed among the PDCD4 knockdown Huh7 cells with both p2 and k603 siRNA than there were negative control cells (Figures 7B,C right panels). Morphological changes were observed in the PDCD4 knockdown cells of all the three hepatoma cell lines, and those cells were frequently positive for β-galactosidase staining (Figure 7A).

### p21 Knockdown Cells Resisted the Down-Regulation of Rb Phosphorylation, CDKs and Induction of Senescence by PDCD4 Knockdown

**Figure 8:**
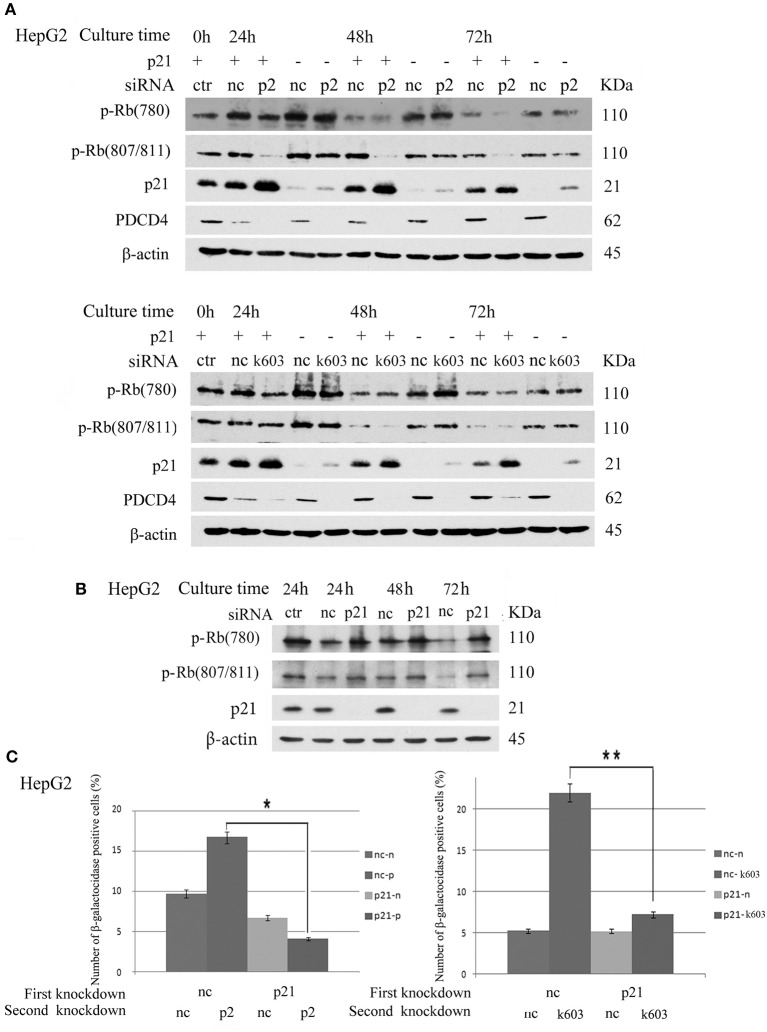
p21 knockdown rescued the down-regulation of Rb phosphorylation and replicative senescence induced by PDCD4 knockdown. **(A)** p21 knockdown suppressed the down-regulation of Rb phosphorylation induced by PDCD4 knockdown. HepG2 cells were first treated with negative control siRNA (nc) or p21-specific siRNA (p21). After culturing for 24 h, each cell sample was then treated with negative control siRNA (nc), PDCD4-specific p2 siRNA (p2, Upper) or k603 siRNA (k603, Lower). The cells were then cultured for a further 24, 48, or 72 h and then subjected to a Western blotting analysis using anti-p-Rb (780), anti-p-Rb (807/811), anti-p21, anti-PDCD4 and anti-β-actin antibodies. **(B)** p21 knockdown up-regulated Rb-phosphorylation. HepG2 cells were treated with negative control siRNA (nc) or p21-specific siRNA (p21) and subjected to a Western blotting analysis with anti-p-Rb (780), anti-p-Rb (807/811), anti-p21 and anti-β-actin antibodies after culturing for the times indicated in the figures. **(C)** p21 knockdown suppressed the PDCD4 knockdown-induced replicative senescence. (Left panel) HepG2 cells were first treated with negative control siRNA (nc) or p21-specific siRNA (p21). After culturing for 24 h, both the nc and p21 knockdown cells were again treated with either negative control siRNA (nc-nc and p21-nc) or PDCD4-specific p2 siRNA (nc-p2 and p21-p2). (Right panel) HepG2 cells were treated the same as in the left panel using PDCD4-specific k603 siRNA. The cells were then fixed and stained with the β-galactosidase assay kit after culturing for an additional 96 h. The galactosidase-positive cell numbers represent the average cell number of three different fields. Thousands of cells were counted in each field. The experiments were repeated twice, and similar results were obtained each time. *t*-test: *p < 0.05; **p < 0.005.

The data suggested that p21 up-regulation might be an important course in senescence induced by PDCD4 knockdown. Therefore, we explored whether or not cells with p21 knockdown resist the down-regulation of Rb phosphorylation or senescence induced by PDCD4 knockdown. As shown in Figure 8A, p21 knockdown rescued Rb phosphorylation induced by PDCD4 knockdown mediated with p2 (upper panel) and k603 (lower panel) siRNA in HepG2 cells. Similar results were also observed in Huh7 cells (Figure S6). CDK1 expression was down-regulated in HepG2 and Huh7 cells, and up-regulated in Hep3B cells by PDCD4 knockdown (Figure 2). p21 knockdown clearly rescued the modulation of CDK1 expression induced by PDCD4 knockdown in all of HepG2, Huh7, and Hep3B cells, while the recovery of CDK2, CDK4, and CDK6 was also observed but not clear (Figure S6). And the accumulation of cell population in pre-G1 phase induced by PDCD4 knockdown was also suppressed by p21 knockdown in three kinds of cells (Figure S7). p21 knockdown itself up-regulated Rb phosphorylation (Figure 8B). To test the effect of p21 knockdown on the induction of senescence by PDCD4 knockdown, HepG2 cells with p21 knockdown were treated with the PDCD4-specific siRNAs p2 and k603. As shown in Figure 8C, the number of β-galactosidase-positive cells among the PDCD4 knockdown cells was significantly reduced in the p21 knockdown cells compared with the negative control cells with both p2 and k603 siRNAs (Figure 8C, left panel and right panel, respectively). Due to the expression of p27 was increased after PDCD4 knockdown in Hep3B cells, we asked whether p27 plays an key role in cell cycle suppression after PDCD4 knockdown. However, we found that p27 knockdown did not alter PDCD4 knockdown-induced changes of CDKs in Hep3B cells. (Figure S8).

## Discussion

In this study, we showed that siRNA-mediated PDCD4 knockdown induced the suppression of cell growth by inhibiting the phosphorylation of retinoblastoma protein (Rb) by down-regulating Rb and CDKs, which phosphorylate Rb in hepatoma cell lines. During cell cycle progression, the cyclin-CDK complex can be regulated by the binding of CDK inhibitor proteins (CKIs) (34), which include two families: Ink4 and Cip/Kip. The Ink4 family includes four proteins (p15, p16, p18, and p19 that interact with CDK4/6. The Cip/Kip family, includes p21, p27, and p57, which inactivate CDK4/6, CDK1 and CDK2. Rb is a central regulator of cell cycle progression. In cell cycle regulation, Rb protein binds to E2F and arrests cell cycle progression (35). Rb phosphorylation mediated by CDKs, such as cyclin D1-CDK4/6 and cyclin E-CDK2, releases E2F, which activates genes required for cell proliferation (36).

We have shown that Rb phosphorylation at both S807/811 and S780 was down-regulated, accompanied by the down-regulation of Rb and CDKs and up-regulation of their inhibitor p21 due to PDCD4 silencing in HepG2 and Huh7 cells. Interestingly, in Hep3B cells lacking p53 and Rb (30), the expression patterns of CDKs in the PDCD4 knockdown cells differed from those in HepG2 and Huh7 cells. G1-S phase regulators CDK2, CDK4, and CDK6 were not significantly modulated but the G2-M phase regulator CDK1 was significantly up-regulated by PDCD4 knockdown in Hep3B cells. At present, whether or not the differences in the CDK expression patterns depend on the presence of p53 and Rb genes is unclear.

p53 is the most common protein involved in carcinogenesis and is activated in response to DNA damage, inducing cell cycle arrest to permit either DNA repair or apoptosis (37). Our data showed that the p21 levels were up-regulated soon after PDCD4 knockdown in hepatoma cells, including Hep3B cells, which are p53-deficient. These results suggest that PDCD4 knockdown may up-regulate the expression of p21 via not only p53 but also the activation of a p53-independent pathway (38, 39). Cells are arrested at the G0-G1phase when p21 is up-regulated by DNA damage, stress and reagents (38, 39). Our FACS data showed that PDCD4 knockdown cells were not arrested at any specific phase of the cell cycle, suggesting that PDCD4 may control not only the Rb functions but also other factors involved in cell cycle progression.

Accumulating data now show that PDCD4 interacts with many factors and modulates the activity of those factors or signaling pathways. It was initially reported that PDCD4 interacts with the eukaryotic translation initiation factor eIF4A and inhibits cap-dependent translation (16). PDCD4 modulates the transcription of specific genes that interact with some transcription factors, such as c-Jun, Sp 1 and Twist 1 (40–42). Daxx, a scaffold protein with roles in a number of different processes, has been shown to be an interactive partner of PDCD4 that disrupts the Daxx-Hipk 2 interaction and thereby inhibits p53 phosphorylation at serine 46 via Hipk 2 (43). Our present data showed that PDCD4 knockdown suppressed Rb phosphorylation and induced cellular senescence, at least in part by up-regulating the p21 expression in a p53-independent manner. These results suggest that PDCD4 might control the p21 expression by interacting with factors involved in the control of the p21 expression. Our data showed that PDCD4 knockdown up-regulated the p21 mRNA levels, findings that concur with those of previous reports (26). Because the promoter region of p21 gene contains Sp1 elements, PDCD4 might directly suppress p21 expression at least partly interacting with Sp1 (44).

Previous studies from our group have shown that the PDCD4 over-expression induced by TGF-β1 led to hepatoma cell apoptosis (6). Apoptosis is a fundamental process that controls cell death and is required for the regulation of the cell number in proliferating tissues. Several regulators of the cell cycle are involved in apoptosis (45, 46). We hypothesized that the knockdown of PDCD4 might induce apoptosis in hepatoma cells via the activation of p53 (47), as our present data show that PDCD4 knockdown activates the caspase cascade and induces apoptosis, which was revealed by FACS and TUNEL assays in HepG2 cells containing wild-type p53, while few apoptotic cells were found in Hep3B with null-p53. In Huh7 cells with mutant p53, many apoptotic cells were observed at Day 5 after PDCD4 knockdown. Therefore, wild-type p53-mediated apoptosis might cause cell death in PDCD4 knockdown cells. p53 phosphorylation at S46 is important for the induction of apoptosis via p53 (43, 48), but we did not observe endogenous levels of p53 S46 phosphorylation in the PDCD4 knockdown cells. However, a reduction in the cell number was also observed in the p53-deficient Hep3B cells after PDCD4 knockdown. Therefore, different cell death pathways independent of p53 might be activated in the PDCD4 knockdown Hep3B cells.

Recent studies have revealed that there are several types of cell death mechanisms other than apoptosis and necrosis in a context-dependent manner (49, 50). Chronic p53-independent p21 expression reportedly caused genomic instability by deregulating replication licensing genes (51). In this context, the p53 checkpoint works to limit re-replication by eliminating re-replicating cells through apoptosis in cells with functional p53 (52), although re-replicating cells accumulate and eventually lead to genomic instability in cells with inactive p53 (51). These results suggest that the DNA loss caused by genomic instability might occur in PDCD4 knockdown Hep3B cells with null p53. Further investigations will be required to determine the mechanisms underlying the cell death in Huh7 and Hep3B cells with PDCD4 knockdown

The growth arrest of a cell is a typical feature of senescence. Cellular senescence was first described as a result of replicative exhaustion, as cells have a finite potential for division; it also occurs as a part of embryonic development, wound-healing, responses to cellular stresses and barriers to carcinogenesis (53–56). Senescent cells are morphologically characterized by an increased cell size, flat shape and positive-staining for β-galactosidase (57) and show cell cycle arrest that is principally regulated by p53 and Rb and accompanied by the up-regulation of tumor suppressor genes, including CDKIs, and the down-regulation of cell-cycle promoting genes, such as CDKs. Furthermore, senescent cells also secrete inflammatory cytokines and growth factors as part of the senescence-associated secretory phenotype (SASP), which might play a role in promoting cancer (54, 56, 58).

Interestingly, in addition to growth suppression, PDCD4 knockdown also induced morphological changes that are characteristic of cellular senescence. Our data showed that PDCD4 knockdown increased the number of β-galactosidase-positive cells, up-regulated the p21 levels and down-regulated the CDKs expression, supporting the induction of cellular senescence in hepatoma cells after PDCD4 knockdown. Because the primary role of cellular senescence is considered to be cancer prevention, the induction of senescence may be useful for the treatment of cancer (59). Indeed, several chemotherapeutic drugs and radiation are known to be able to induce senescence in tumor cells (60, 61), including inhibitors of CDK4, aurora kinase B (AURKB), casein kinase 2 (CK2), and sunitinib, a known multi-targeted receptor tyrosine kinase inhibitor (56, 62). Therefore, PDCD4 may be a target for the treatment of liver cancer, although further studies both *in vitro* and *in vivo* are required.

However, conflicting results regarding PDCD4 knockdown have been reported. PDCD4 knockout mice develop normally to adulthood, indicating that PDCD4 is not indispensable for development and cell proliferation under normal physiological conditions (29). It has been reported that PDCD4 knockdown stimulates cell proliferation by up-regulating the cyclin D1 expression in HT29 colon carcinoma cells and keratinocytes (28, 63). In our experiments, the significant up-regulation of cyclin D1 was not observed; in fact, its expression was actually decreased in PDCD4 knockdown hepatoma cells. These discrepant responses to PDCD4 knockdown among cell lines may be due to differences in cell type and experimental settings. The mechanism by which PDCD4 knockdown differently affects the behavior of cells needs to be clarified in the future.

In conclusion, PDCD4 knockdown induced cell growth arrest and cellular senescence by up-regulating p21 and down-regulating CDKs, irrespective of the p53 status, in human hepatoma cells. Our results indicate that PDCD4 plays important roles in cell cycle regulation and the induction of cellular senescence in human hepatoma cells and may be a potential target in antineoplastic therapies.

## Author Contributions

IO and SM devised the study and designed the experiments. JG, JX, TK, MK, HT, and SM performed the experiments. JG, IO, JX, TK, MK, HT, and SM analyzed the data. IO, KenA, SM, and KeiA provided materials and scientific support for the study. JG, IO, and SM wrote the manuscript. All authors have read and approved the final manuscript.

### Conflict of Interest Statement

The authors declare that the research was conducted in the absence of any commercial or financial relationships that could be construed as a potential conflict of interest.

## Supplementary Material

Figure S1 K603 siRNA-mediated PDCD4 knockdown modulated the Rb expression, Rb-phosphorylation and expression of CDKs in **(A)** HepG2, **(B)** Huh7, and **(C)** Hep3B cells, similar to p2 siRNA (Figure 2). **(D)** The modulation of Rb-phosphorylation in p1 and p2 siRNA mediated PDCD4 knockdown cells. Experiments were performed as described in Figure 2.

Figure S2 K603 siRNA-mediated PDCD4 knockdown up-regulated p21 protein **(A-C)** and mRNA **(D)** in HepG2, Huh7 and Hep3B cells. **(E)** p2 siRNA-mediated PDCD4 knockdown up-regulated the p21 expression in p53 knockdown HepG2 cells, similar to p2 siRNA-treated Huh7 cells (Figure 3E). Experiments were performed as described in Figure 3.

Figure S3 The modulation of the p27 protein **(A)** and mRNA **(B)** expression induced by K603 siRNA-mediated PDCD4 knockdown. The results were similar to those obtained with p2 siRNA (Figure 4).

Figure S4 The modulation of the INK4 p16 and p18 expression by k603 siRNA-mediated PDCD4 knockdown. A Western blot analysis (Left) and diagrams of the p16 expression (Right) obtained from the Western blot of HepG2 **(A)** and Hep3B **(B)**. Experiments were performed as described in Figure 5 using k603 siRNA. The p16 expression was not change significantly by k603 siRNA treatment in both HepG2 and Hep3B cells. The p18 protein **(C)** and mRNA **(D)** levels were not changed or a little down-regulated by PDCD4 knockdown in HepG2, Huh7 cells and Hep3B cells. Significant p-values were not obtained by a t-test between nc and k603 siRNA treatments.

Figure S5 Apoptosis induced by k603 siRNA-mediated PDCD4 knockdown in HepG2, Huh7, and Hep3B cells. **(A)** A caspase assay at (1) 24, 48, and 72 h and (2) 5 days' culture in HepG2, Huh7, and Hep3B cells. **(B)** A TUNEL assay in HepG2, Huh7, and Hep3B cells. **(C)** A FACS analysis in HepG2, Huh7, and Hep3B cells. All experiments were performed using k603 siRNA, as described in Figure 6.

Figure S6 p21 knockdown rescued the down-regulation of p-Rb and CDKs induced by p2 siRNA mediated PDCD4 knockdown in HepG2, Huh7, and Hep3B cells. Experiments were performed as described in Figure 8. (nc, negative control siRNA; p2, PDCD4-specific p2 siRNA). p21 knockdown clearly rescued the CDK1 modulation induced by PDCD4 knockdown in all of HepG2, Huh7, and Hep3B cells, but that of CDK2, CDK4, and CDK6 was not clear. Similar results were obtained by using k603 siRNA (data not shown).

Figure S7 p21 knockdown reduced the accumulation of cell population in pre-G1 phase induced by PDCD4 knockdown. The cells were first treated with negative control siRNA (nc) or p21-specific siRNA (p21). After culturing for 24 h, each cell sample was then treated with negative control siRNA (nc), PDCD4-specific p2 siRNA (p2) or k603 siRNA (k). The cells were then cultured for a further 72, 96, or 120 h and then subjected to FACS analysis. (nn, negative control and negative control siRNA treated; np2 or nk603, negative control and PDCD4-specific p2 or negative control and k603 siRNA-treated; p21p2 or p21k603, p21-specific siRNA and PDCD4-specific p2 or p21-specific siRNA and k603 siRNA-treated; p21nc, p21-specific siRNA and negative control siRNA treated.) The experiments were independently repeated at least three times, and the data represent the mean ± SD obtained from the experiments. *t*-test: ^*^*p* < 0.05; ^**^*p* < 0.005.

Figure S8 p27 knockdown did not alter PDCD4 knockdown-induced changes of cell cycle regulators in Hep3B cells. (nc, negative control siRNA; p2, PDCD4-specific p2 siRNA). The cells were first treated with negative control siRNA (nc) or p27-specific siRNA (p27). After culturing for 24 h, each cell sample was then treated with negative control siRNA (nc) or PDCD4-specific p2 siRNA (p2). The cells were then cultured for a further 48 or 72 h and then subjected to a Western blotting analysis.
