# Supplementary figures and images for: PDCD4 Knockdown Induces Senescence in Hepatoma Cells by Up-Regulating the p21 Expression
Source: Front Oncol. 2019 Jan 9;8:661. doi: 10.3389/fonc.2018.00661 (PMC6334536; doi:10.3389/fonc.2018.00661)

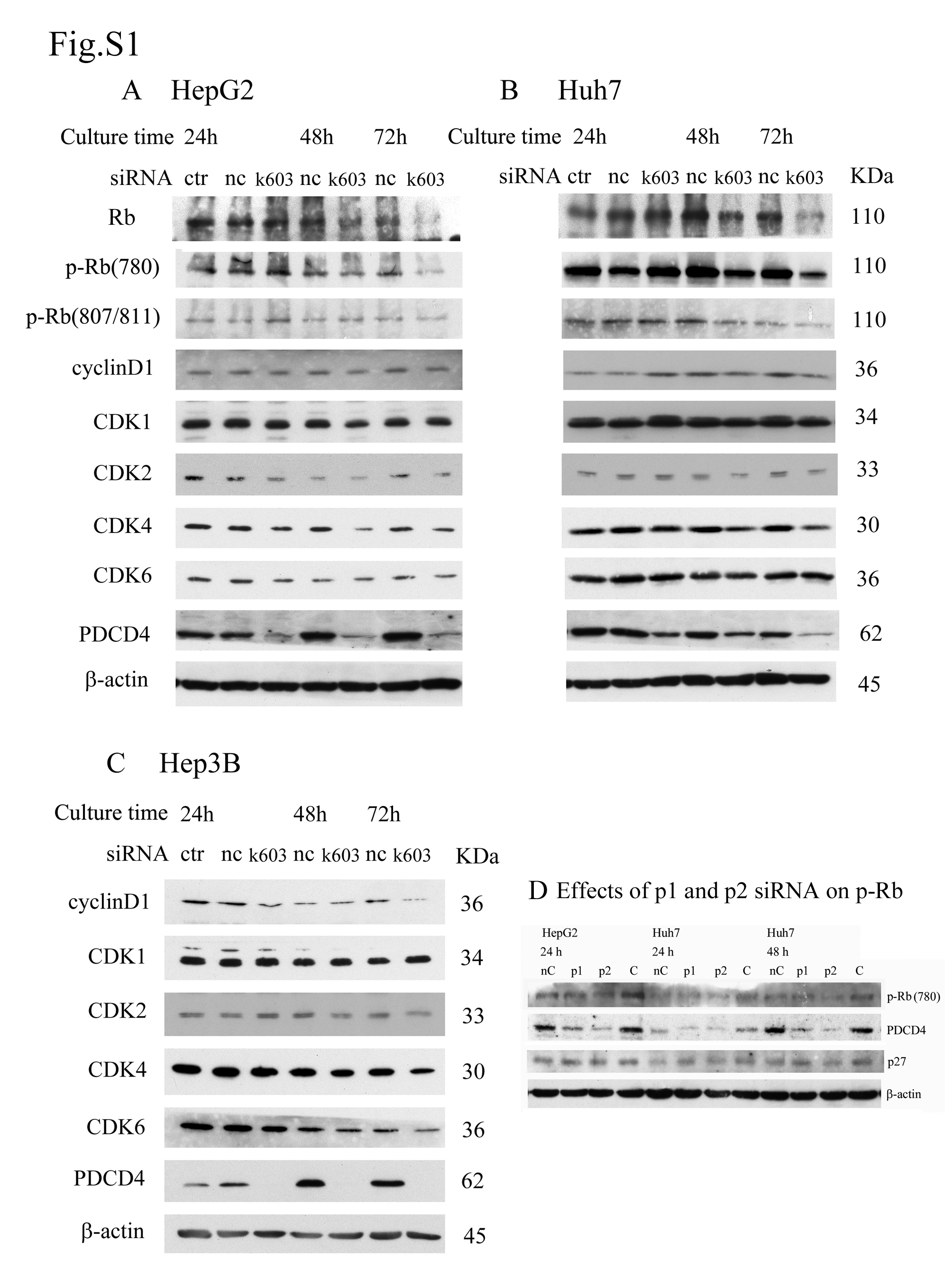

Supplement: Figure S1 — K603 siRNA-mediated PDCD4 knockdown modulated the Rb expression, Rb-phosphorylation and expression of CDKs in (A) HepG2, (B) Huh7, and (C) Hep3B cells, similar to p2 siRNA (Figure 2). (D) The modulation of Rb-phosphorylation in p1 and p2 siRNA mediated PDCD4 knockdown cells. Experiments were performed as described in Figure 2. [file Image_1.TIF]

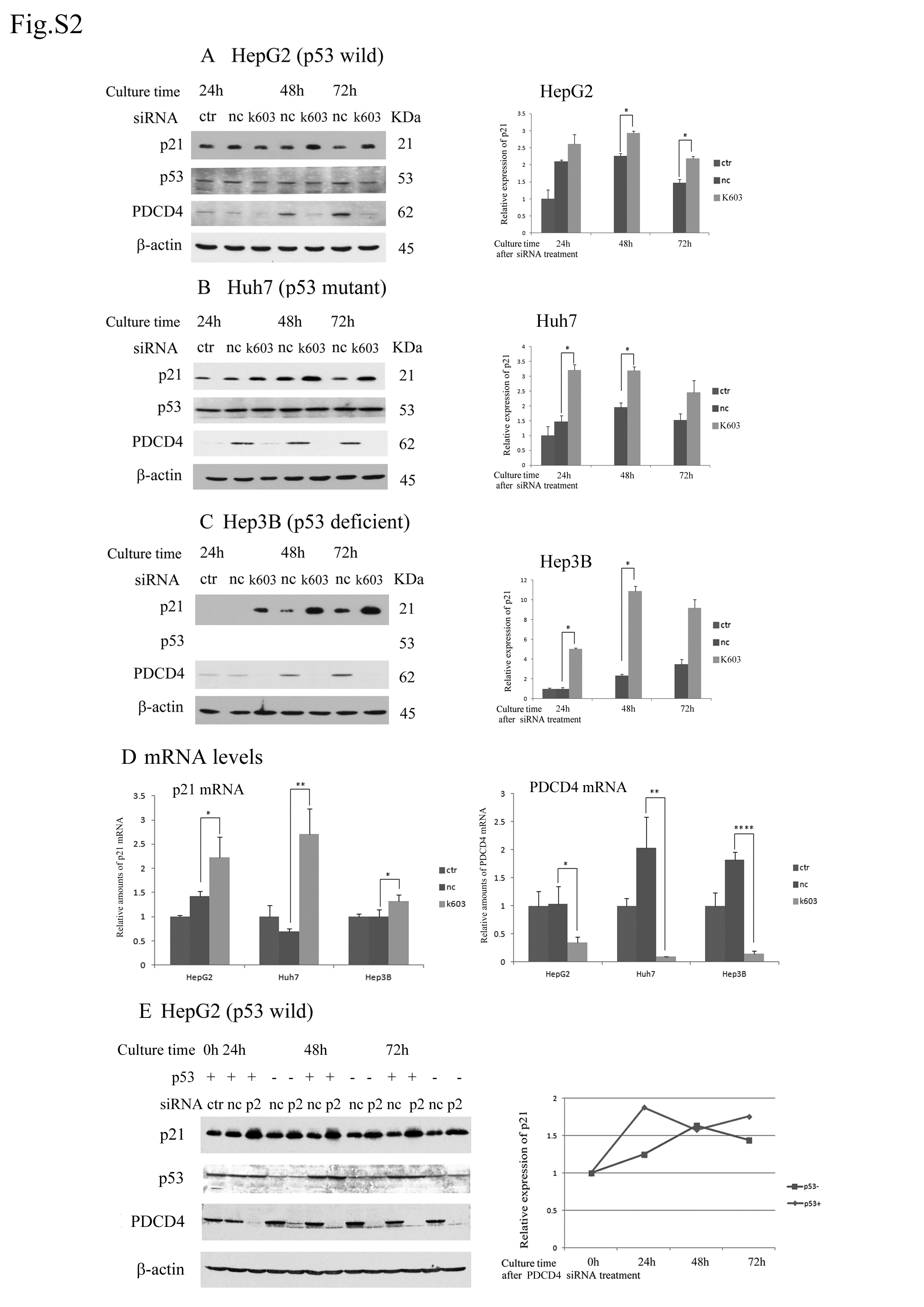

Supplement: Figure S2 — K603 siRNA-mediated PDCD4 knockdown up-regulated p21 protein (A-C) and mRNA (D) in HepG2, Huh7 and Hep3B cells. (E) p2 siRNA-mediated PDCD4 knockdown up-regulated the p21 expression in p53 knockdown HepG2 cells, similar to p2 siRNA-treated Huh7 cells (Figure 3E). Experiments were performed as described in Figure 3. [file Image_2.TIF]

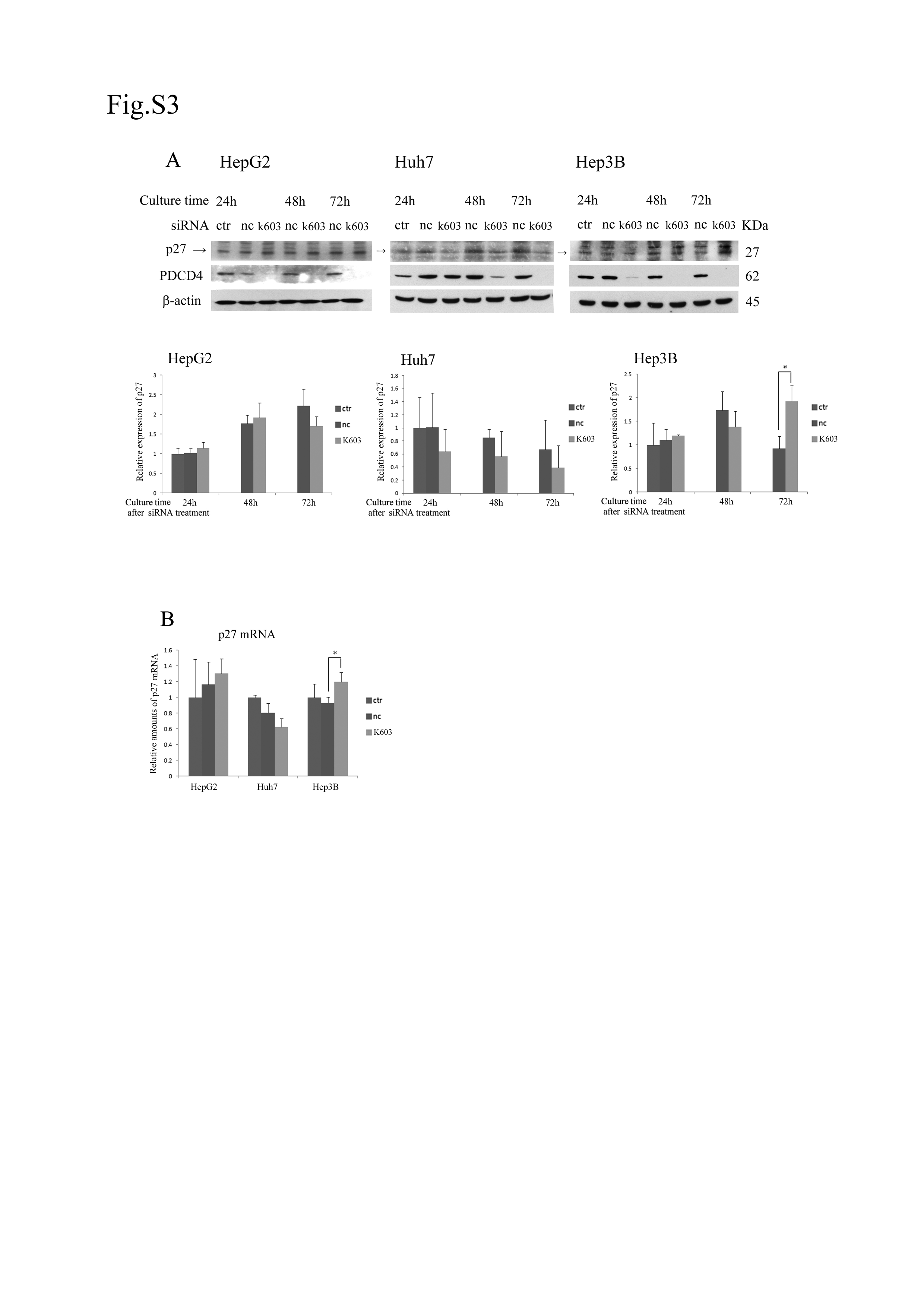

Supplement: Figure S3 — The modulation of the p27 protein (A) and mRNA (B) expression induced by K603 siRNA-mediated PDCD4 knockdown. The results were similar to those obtained with p2 siRNA (Figure 4). [file Image_3.TIF]

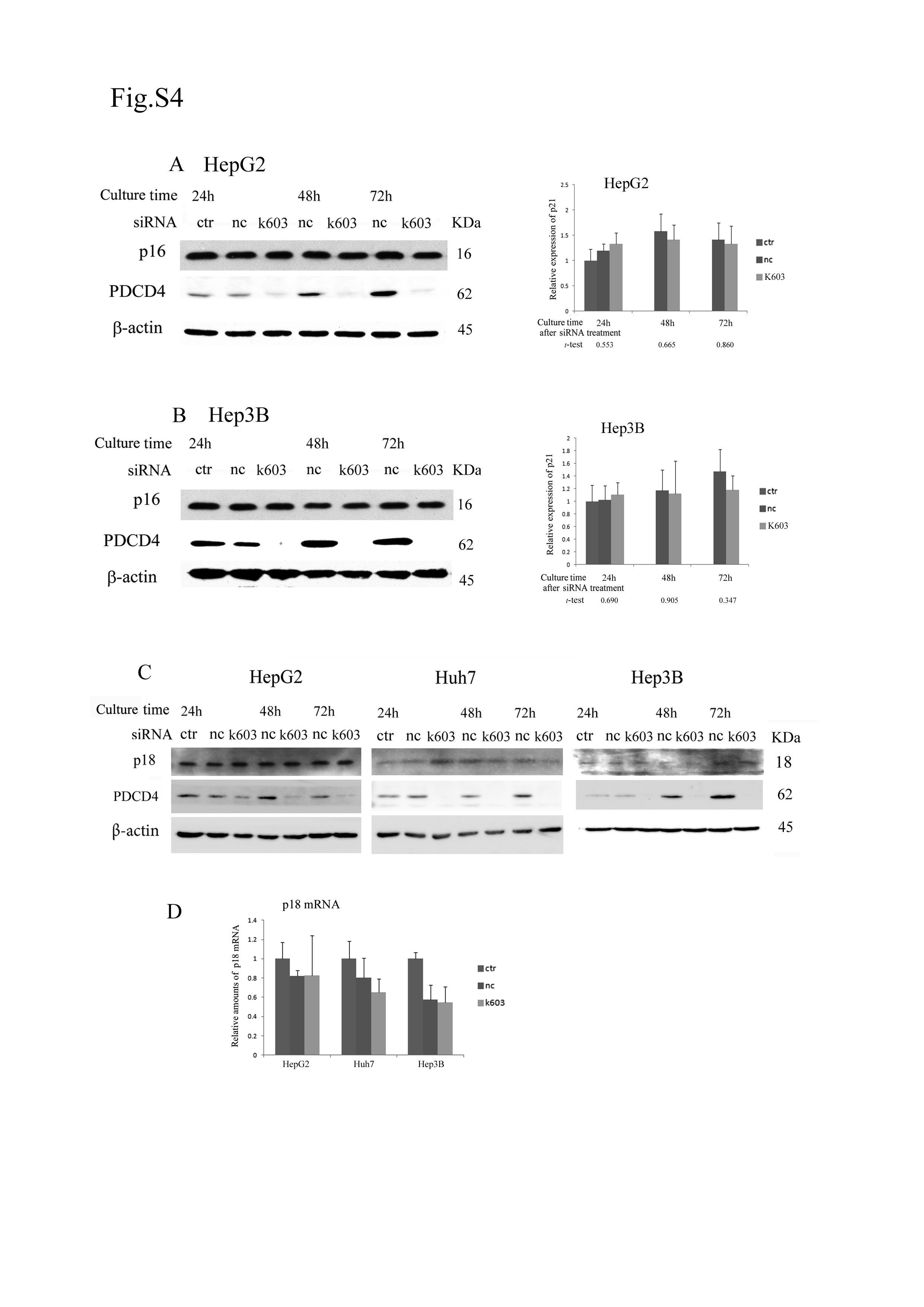

Supplement: Figure S4 — The modulation of the INK4 p16 and p18 expression by k603 siRNA-mediated PDCD4 knockdown. A Western blot analysis (Left) and diagrams of the p16 expression (Right) obtained from the Western blot of HepG2 (A) and Hep3B (B). Experiments were performed as described in Figure 5 using k603 siRNA. The p16 expression was not change significantly by k603 siRNA treatment in both HepG2 and Hep3B cells. The p18 protein (C) and mRNA (D) levels were not changed or a little down-regulated by PDCD4 knockdown in HepG2, Huh7 cells and Hep3B cells. Significant p-values were not obtained by a t-test between nc and k603 siRNA treatments. [file Image_4.TIF]

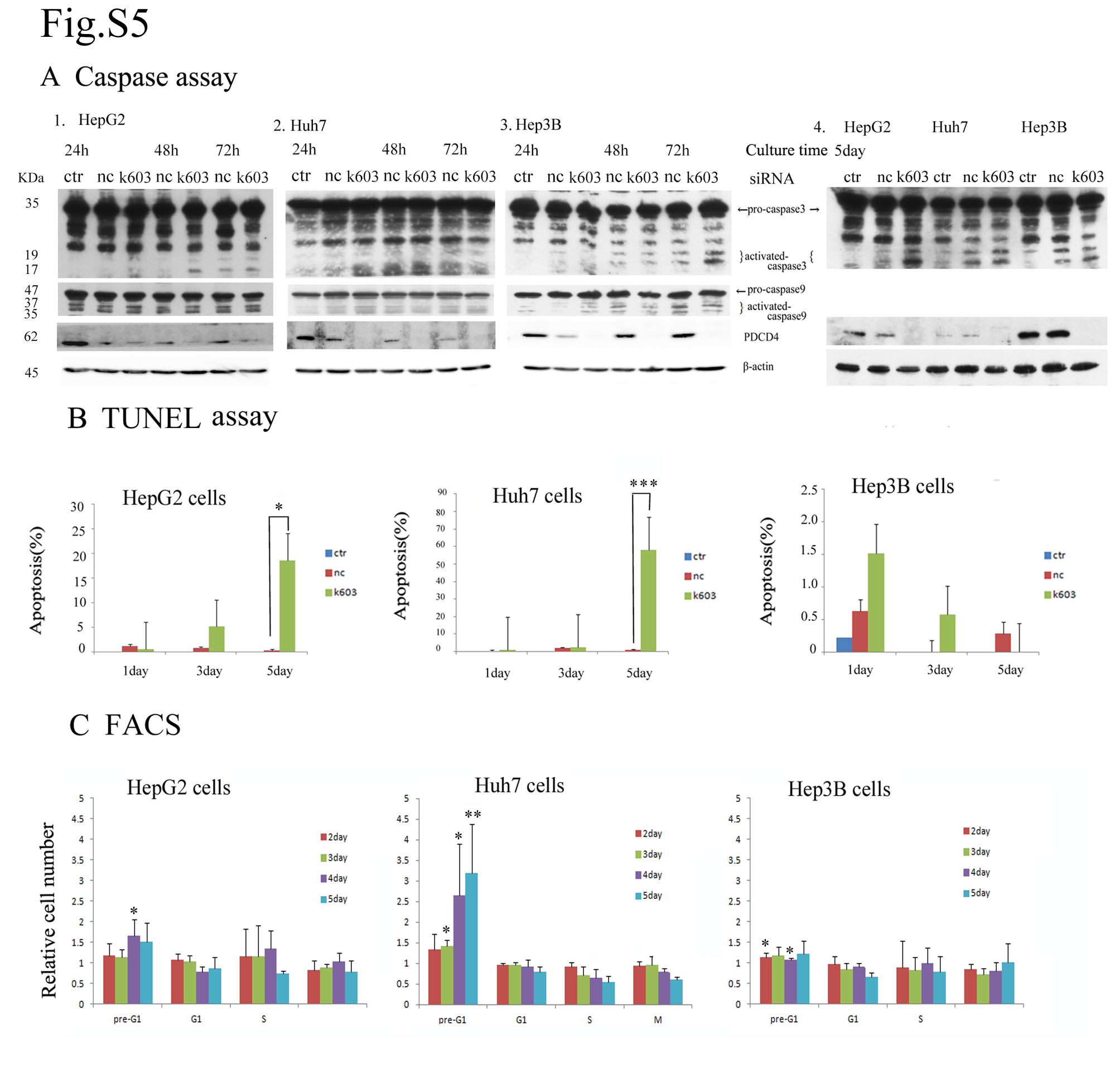

Supplement: Figure S5 — Apoptosis induced by k603 siRNA-mediated PDCD4 knockdown in HepG2, Huh7, and Hep3B cells. (A) A caspase assay at (1) 24, 48, and 72 h and (2) 5 days' culture in HepG2, Huh7, and Hep3B cells. (B) A TUNEL assay in HepG2, Huh7, and Hep3B cells. (C) A FACS analysis in HepG2, Huh7, and Hep3B cells. All experiments were performed using k603 siRNA, as described in Figure 6. [file Image_5.TIF]

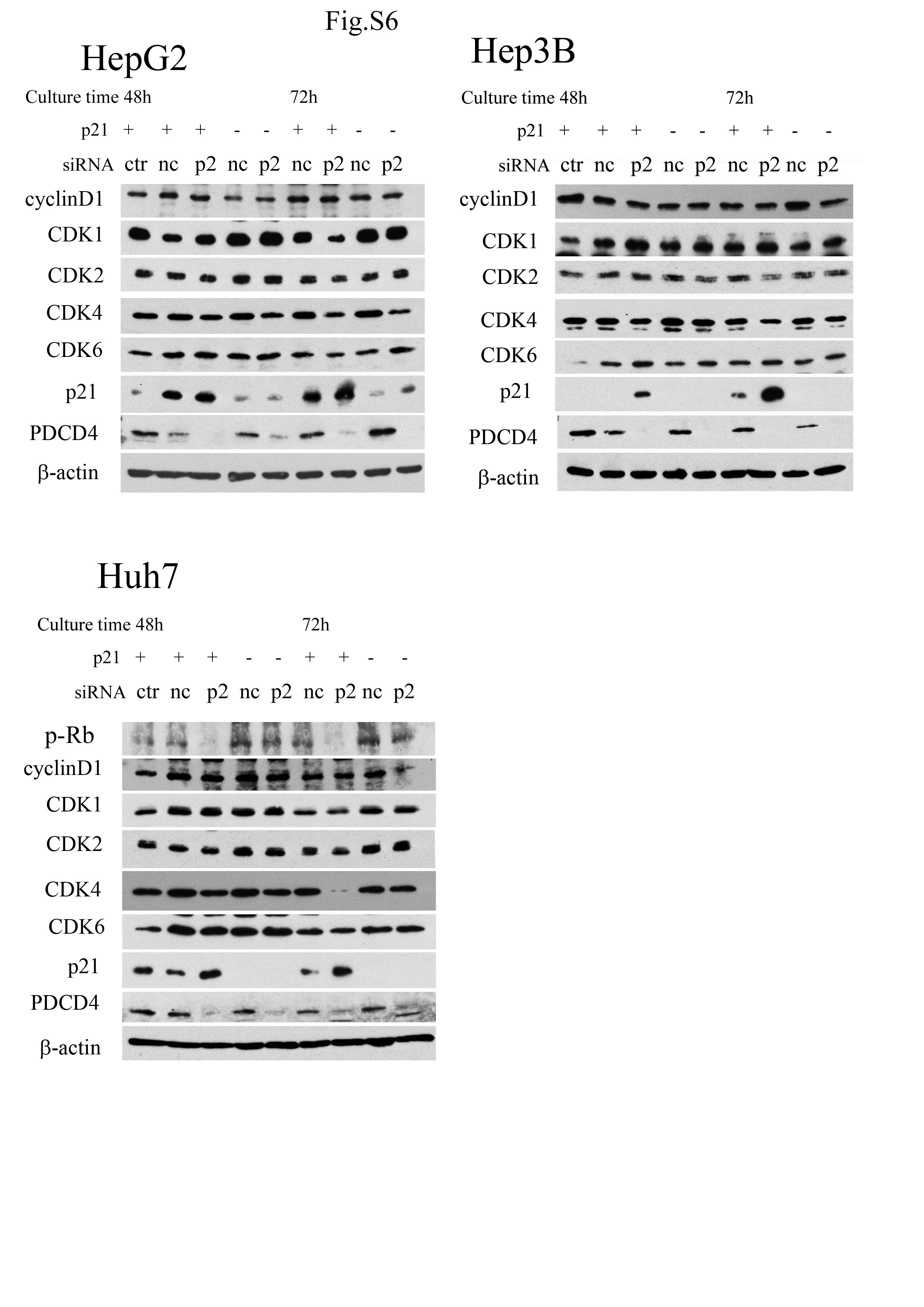

Supplement: Figure S6 — p21 knockdown rescued the down-regulation of p-Rb and CDKs induced by p2 siRNA mediated PDCD4 knockdown in HepG2, Huh7, and Hep3B cells. Experiments were performed as described in Figure 8. (nc, negative control siRNA; p2, PDCD4-specific p2 siRNA). p21 knockdown clearly rescued the CDK1 modulation induced by PDCD4 knockdown in all of HepG2, Huh7, and Hep3B cells, but that of CDK2, CDK4, and CDK6 was not clear. Similar results were obtained by using k603 siRNA (data not shown). [file Image_6.TIF]

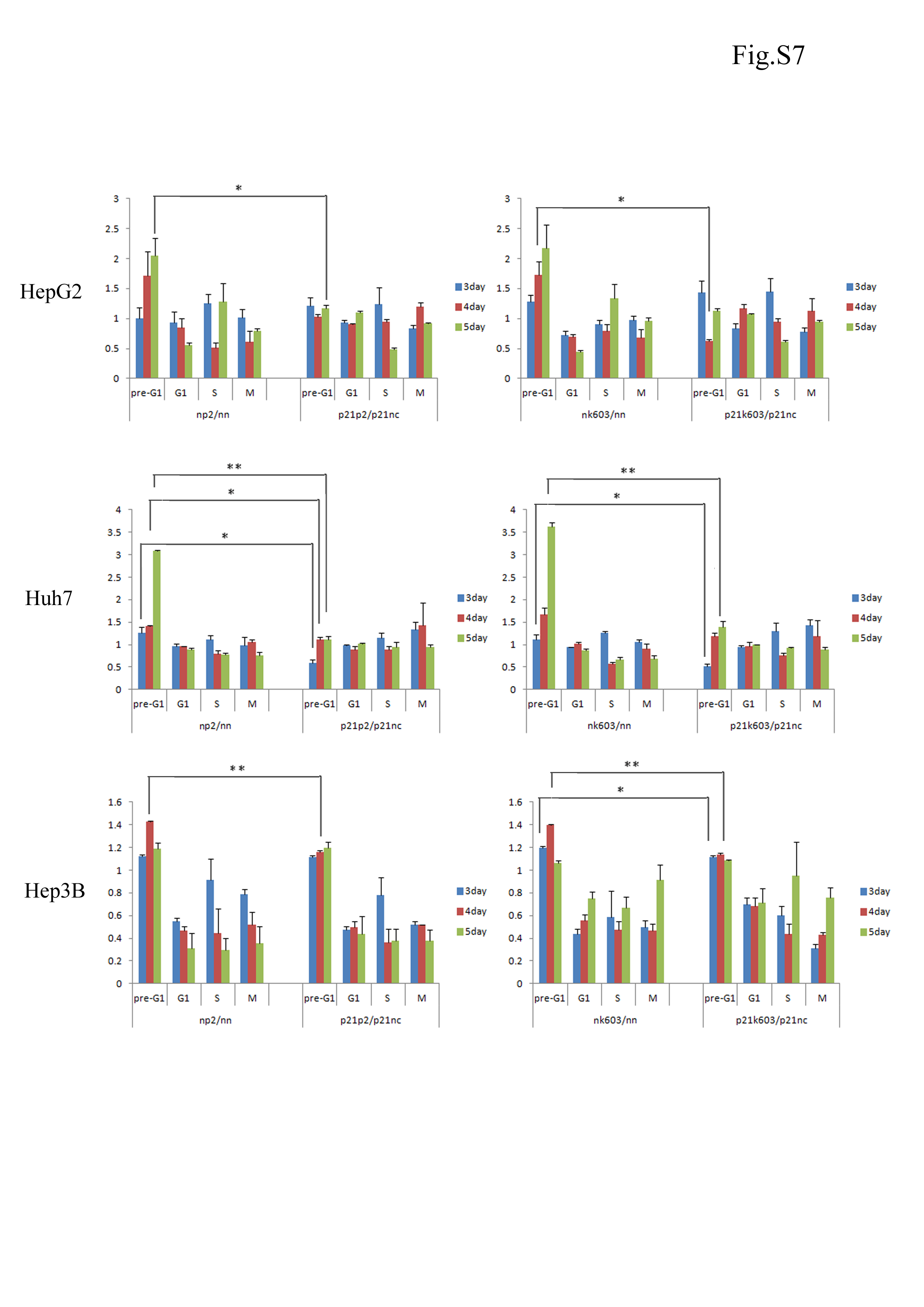

Supplement: Figure S7 — p21 knockdown reduced the accumulation of cell population in pre-G1 phase induced by PDCD4 knockdown. The cells were first treated with negative control siRNA (nc) or p21-specific siRNA (p21). After culturing for 24 h, each cell sample was then treated with negative control siRNA (nc), PDCD4-specific p2 siRNA (p2) or k603 siRNA (k). The cells were then cultured for a further 72, 96, or 120 h and then subjected to FACS analysis. (nn, negative control and negative control siRNA treated; np2 or nk603, negative control and PDCD4-specific p2 or negative control and k603 siRNA-treated; p21p2 or p21k603, p21-specific siRNA and PDCD4-specific p2 or p21-specific siRNA and k603 siRNA-treated; p21nc, p21-specific siRNA and negative control siRNA treated.) The experiments were independently repeated at least three times, and the data represent the mean ± SD obtained from the experiments. t-test: *p < 0.05; **p < 0.005. [file Image_7.TIF]

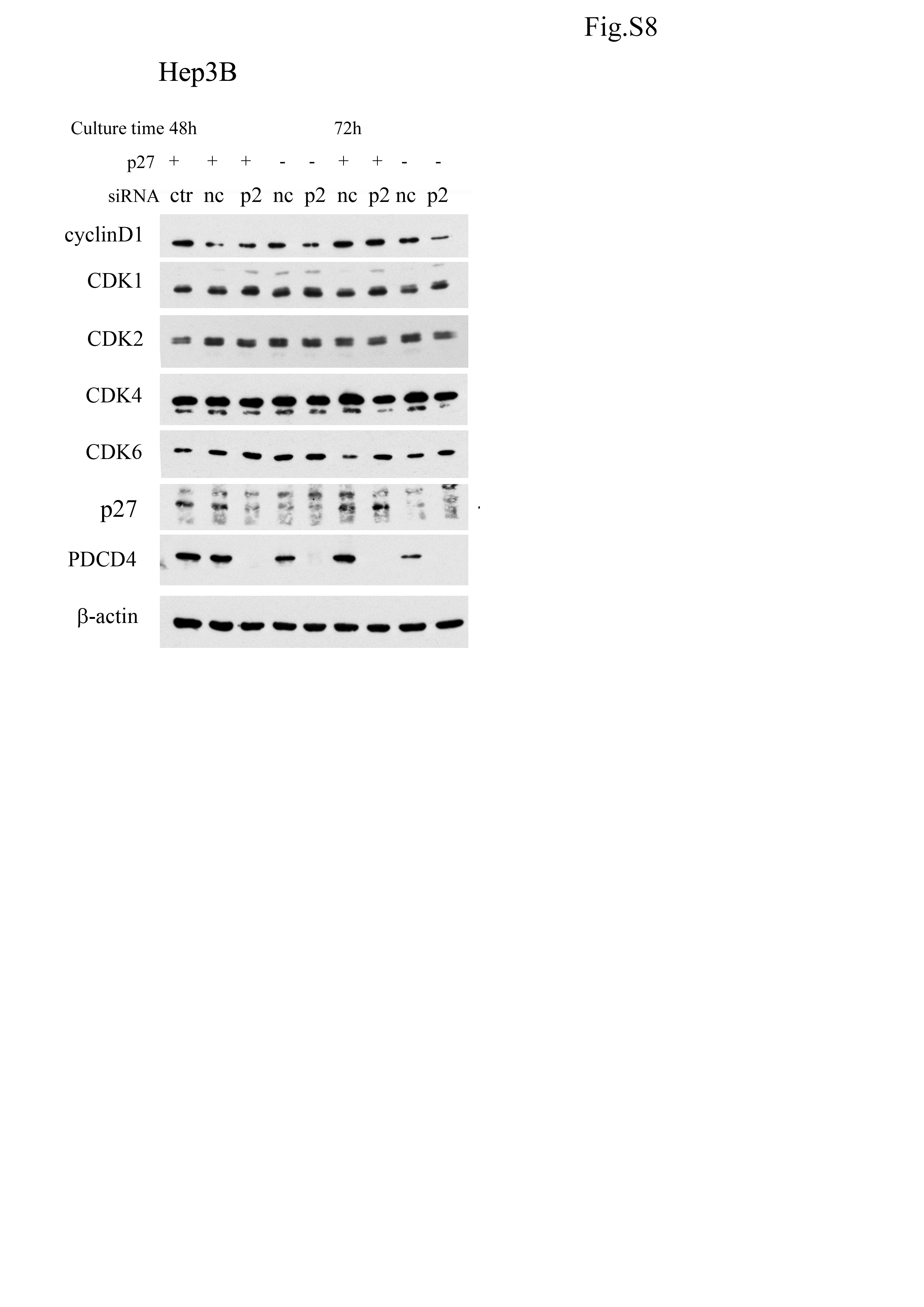

Supplement: Figure S8 — p27 knockdown did not alter PDCD4 knockdown-induced changes of cell cycle regulators in Hep3B cells. (nc, negative control siRNA; p2, PDCD4-specific p2 siRNA). The cells were first treated with negative control siRNA (nc) or p27-specific siRNA (p27). After culturing for 24 h, each cell sample was then treated with negative control siRNA (nc) or PDCD4-specific p2 siRNA (p2). The cells were then cultured for a further 48 or 72 h and then subjected to a Western blotting analysis. [file Image_8.TIF]
